# The Complete Mitochondrial Genome of *Liobagrus huaiheensis* (Teleostei: Siluriformes: Amblycipitidae): Characterization, Phylogenetic Placement, and Insights into Genetic Diversity

**DOI:** 10.3390/genes16080977

**Published:** 2025-08-19

**Authors:** Chaoqun Su, Chenxi Tan, Liangjie Zhao, Jiahui Liu, Xusheng Guo, Gaoyou Yao, Weizhao Zhang, Tiezhu Yang

**Affiliations:** 1 School of Fisheries, Xinyang Agriculture and Forestry University, Xinyang 464000, China; cqsu@xyafu.edu.cn (C.S.); cxtan@xyafu.edu.cn (C.T.); 2016210001@xyafu.edu.com (L.Z.); 2021210005@xyafu.edu.com (J.L.); gxs1968@xyafu.edu.cn (X.G.); 2023210004@xyafu.edu.com (G.Y.); 15264679296@163.com (W.Z.); 2 Fishery Biological Engineering Technology Research Center of Henan Province, Xinyang 464000, China

**Keywords:** *Liobagrus huaiheensis*, mitochondrial genome, phylogenetic analysis, genetic diversity

## Abstract

**Background/Objectives**: *Liobagrus huaiheensis*, an endemic fish in the Huaihe River basin, is a newly described species with limited molecular genetic research, hindering understanding of its evolutionary status, population structure, and genetic diversity. This study aimed to characterize its complete mitochondrial genome, clarify its phylogenetic position within *Liobagrus*, and assess its population genetic diversity. **Methods**: We obtained the complete mitogenome of *L. huaiheensis* (sourced from the Zhugan River) through sequencing, followed by detailed annotation of this genomic sequence. We analyzed its genomic structure, nucleotide composition, codon usage, and base asymmetry. Selection pressure on 13 protein-coding genes (PCGs) was evaluated using Ka/Ks ratios. Phylogenetic trees were generated by means of Bayesian inference (BI) and maximum likelihood (ML), using a dataset composed of 13 protein-coding genes (PCGs) from 37 species. Population genetic diversity was assessed using the *cox1* gene. **Results**: The mitogenome is a 16,512 bp circular molecule encoding 37 genes and one control region, with a conserved structure typical of *Liobagrus*. It has high A + T content (55.74%) with A-preference and C-enrichment. All PCGs undergo purifying selection (Ka/Ks < 1). Phylogenetic analyses revealed *L. huaiheensis* is closest to *L. obesus* (100% support), with *Liobagrus* divided into three clades. The *cox1* gene analysis showed low diversity (Hd = 0.656, π = 0.00171) and neutral evolution. **Conclusions**: This study fills the mitogenome data gap for *L. huaiheensis*, clarifies its evolutionary characteristics and phylogenetic position, and provides a basis for conservation genetics of Huaihe endemic fishes and molecular evolution research on Amblycipitidae.

## 1. Introduction

The genus *Liobagrus* (Amblycipitidae) comprises freshwater catfishes widely distributed in freshwater ecosystems across central and southern China, Japan, and the Korean Peninsula, with their taxonomic status and evolutionary history being focal points of ichthyological research [1]. *L. huaiheensis*, a newly described species in 2021, was initially discovered in the Shiguan River (a tributary of the Huaihe River), and subsequent surveys have expanded its known distribution to the main stream of the Huaihe River and other tributaries (e.g., Zhugan River, Shi River). It represents the only endemic fish among 73 recorded species in the Huaihe River basin [2]. Distinguished by unique morphological traits (e.g., the posterior margin of the pectoral-fin spine is equipped with two to three serrations, while the anal fin contains 15 to 17 rays in total) and its biogeographic position, this species serves as a key taxon linking *Liobagrus* populations in China and the Korean Peninsula [3,4,5].

Mitochondrial genomes (mitogenomes) are valuable molecular markers in phylogenetic reconstruction, population genetics, and species delimitation due to their conserved structure, moderate evolutionary rate, and maternal inheritance [6,7]. Despite progress in the taxonomic study of *Liobagrus* (e.g., morphological classification and distribution mapping), molecular genetic research on *L. huaiheensis* remains limited. The lack of complete mitogenome data for this species hinders insights into its evolutionary status, population structure, and genetic diversity. Additionally, phylogenetic relationships within *Liobagrus* remain unresolved, highlighting the need for mitogenome-based analyses to clarify evolutionary affinities [8].

This research represents the first attempt to determine the complete mitogenome of *L. huaiheensis*, with a subsequent detailed characterization of its genomic features. We systematically analyzed its genomic structure, nucleotide composition, codon usage patterns of protein-coding genes (PCGs), features of ribosomal RNAs (rRNAs) and transfer RNAs (tRNAs), and assessed base asymmetry via AT/GC skew. Furthermore, we conducted selection pressure analyses on 13 PCGs, reconstructed the phylogeny of *Liobagrus* species, and evaluated the genetic diversity of the Huaihe River population. The objectives were to (1) fill the gap in molecular data for *L. huaiheensis* and provide genetic markers for species identification; (2) elucidate the evolutionary characteristics and adaptive selection mechanisms of its mitogenome; and (3) clarify its phylogenetic position within *Liobagrus* to enhance understanding of the genus’ biogeographic evolution. This research will lay a foundation for the conservation genetics of endemic fishes in the Huaihe River and molecular evolution studies of Amblycipitidae.

## 2. Materials and Methods

### 2.1. Sample Collection and Processing

**Figure 1:**
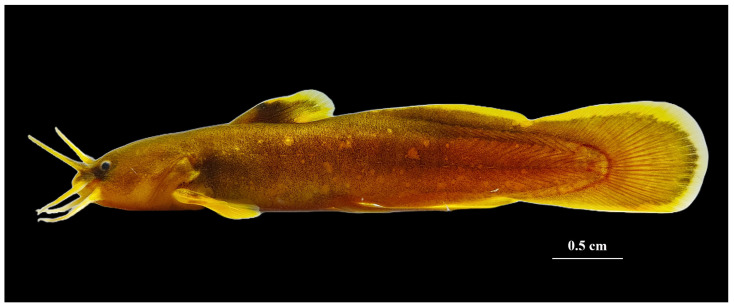
Detailed photograph of *L. huaiheensis* (photographed by Weizhao Zhang).

Adult specimens of *L. huaiheensis* (Figure 1) in this study were collected using cage traps in August 2024 from the Zhugan River, a tributary of the Huaihe River in Luoshan County, Xinyang City, Henan Province (geographic coordinates: 114°37′4″ E, 32°4′21″ N). Samples were initially identified morphologically on-site and immediately preserved in 100% ethanol. Upon returning to the laboratory, specimens were further confirmed as *L. huaiheensis* through morphological examination and stored at −80 °C until DNA extraction. All experimental procedures were conducted in strict compliance with international guidelines governing the care and handling of laboratory animals. Some individuals were prepared as vouchers and deposited in the Herbarium of Xinyang Agriculture and Forestry University under the accession number XYAFU-Mo-240811630.

### 2.2. DNA Extraction, Library Construction, and Sequencing

The Tissue DNA Extraction Kit (DP304, Tiangen Biochemical Technology (Beijing) Co., Ltd., Beijing, China) was employed to extract total genomic DNA from the muscle tissue of *L. huaiheensis*. Subsequently, the purity and concentration of the obtained DNA were determined with a NanoDrop 2000 instrument (Thermo Fisher Scientific, Waltham, MA, USA). DNA libraries were prepared with the TIANSeq Fast DNA Library Prep Kit (Illumina) (NG102-01; Tiangen Biochemical Technology (Beijing) Co., Ltd., Beijing, China). The concentrations of the libraries were measured with a Qubit 4 Fluorometer (Thermo Fisher Scientific, Waltham, MA, USA), and the fragment sizes of the libraries were assessed using an Agilent 2100 Bioanalyzer (Agilent Technologies, Santa Clara, CA, USA). The Illumina NovaSeq 6000 platform (Illumina, San Diego, CA, USA) was then utilized for library sequencing, yielding 150 bp paired-end reads.

### 2.3. Assembly and Annotation of the Mitochondrial Genome

Quality control and filtering of raw sequencing data were performed using Fastp v0.36 [9]. Second-generation sequencing data were assembled with SPAdes v3.15 [10], and gaps in the resulting contigs were filled using GapFiller v 2.1.2 [11]. Base errors and small indels in the assembly were corrected with Pilon v 1.24 [12]. Coding sequence (CDS) boundaries were determined by combining tblastn and genewise v 2.2.0 [13] against a closely related reference genome. Transfer RNA (tRNA) sequences were predicted using MitoFinder v 1.4.2 [14] and tRNAscan-SE 2.0 search server [15], and ribosomal RNA (rRNA) elements were identified via CMsearch [16] against the Rfam database [17]. Additionally, the mitochondrial genome sequence was automatically annotated using the MITOS2 de novo annotation tool on the Galaxy Web Server [18].

### 2.4. Analysis of Mitochondrial Genome Sequence Characteristics

MEGA v 11.0 [19] software was employed to analyze both the base composition and codon usage patterns of the *L. huaiheensis* mitochondrial genome, while the relative synonymous codon usage (RSCU) for each protein-coding gene (PCG) was analyzed using CodonW v 1.4.2 [20]. The A + T skew value was derived using (A% − T%)/(A% + T%), whereas the G + C skew value was calculated with (G% − C%)/(G% + C%) [21]. Substitution rates, encompassing both nonsynonymous (Ka) and synonymous (Ks) rates, among closely related species were determined using KaKs Calculator v3.0 [22].

### 2.5. Phylogenetic Analysis Methods

A total of 37 complete mitochondrial sequences of 37 species from 15 genera of 5 families were downloaded from NCBI data, including 13 species belonging to the genus *Liobagrus* (Table 1). Nucleotide sequences of the 13 protein-coding genes (PCGs) from all species were aligned and format-converted using AliView v 1.2.6 [23]. Information and accession numbers of the species used in this study are listed in Table 1. *Kryptopterus vitreolus* and *Kryptopterus bicirrhis* from the family Siluridae were selected as outgroups for phylogenetic tree construction [24]. PartitionFinder v 2.1.1 was used to identify the most suitable evolutionary models for the analysis [25]. After organizing and concatenating the 13 PCGs’ nucleotide sequences in a specific order, we analyzed phylogenetic relationships using the ML and BI approaches. IQ-TREE v 2.3.6 [26] was used to build the ML tree with 1000 bootstrap replicates. BI analysis was conducted in MrBayes v 3.2.7a [27], with four MCMC chains running for 200,000 generations (sampled every 1000 generations) and a 25% burn-in applied to the initial dataset. The resulting phylogenetic trees were generated and visualized using FigTree v 1.4.4 and Adobe Illustrator 2020.

### 2.6. Population-Level Genetic Diversity Analysis

This research involved the collection and storage of DNA samples from 30 adult *L. huaiheensis* specimens. For the assessment of population genetic diversity, the mitochondrial *cytochrome b* (*cyt b*) gene of *L. huaiheensis* was selected as the molecular marker [44]. Specific primers for the *cox1* gene were used for amplification: Lh-cox1-F (5′-GACTTGAAAAACCACCGTTG-3′) and Lh-cox1-R (5′-CTCCGATCTCCGGATTACAAGAC-3′), targeting a 1150 bp fragment [45,46]. PCR reactions were performed using TaKaRa Taq™ HS Perfect Mix (Cat. No. R300A; Takara Biomedical Technology (Beijing) Co., Ltd., Shiga, Japan) following the manufacturer’s instructions. PCR amplicons were subjected to bidirectional sequencing services provided by Sangon Biotech (Shanghai, China), and sequences were assembled using DNAstar v 7.1.0 software [47]. Population genetic diversity was analyzed using DnaSP v 6.12.03 software [48].

## 3. Results and Discussion

### 3.1. Overall Characteristics of the Mitochondrial Genome of L. huaiheensis

**Figure 2:**
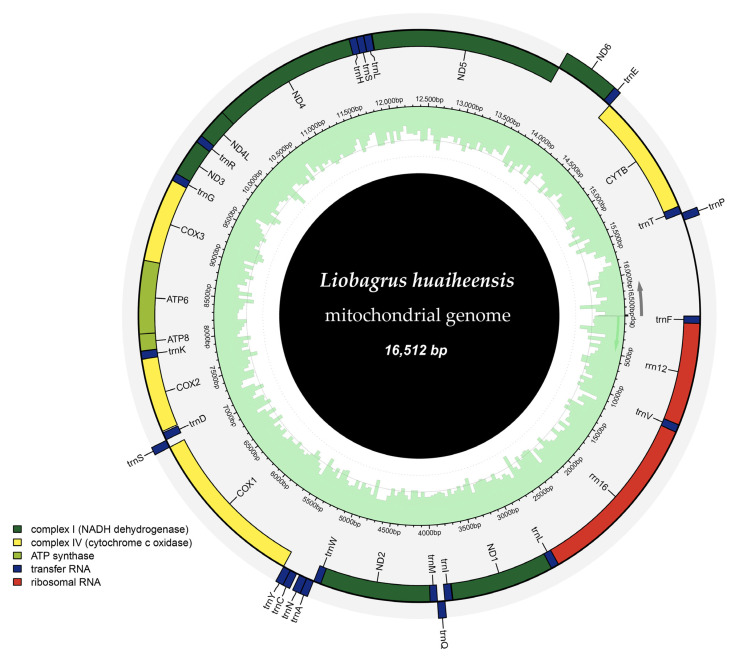
Circos plot of the mitochondrial genome of *L. huaiheensis*. In the outermost gene elements, the inner circle shows genes transcribed in the forward direction, and the outer circle shows genes transcribed in the reverse direction; light green bars in the middle ring indicate the sequencing depth of the corresponding regions.

Following de novo assembly and subsequent annotation of high-throughput sequencing data (with 100% assembly coverage), the mitochondrial genome of *L. huaiheensis* was characterized as a double-stranded circular molecule. This genome has a total length of 16,512 bp and harbors 37 annotated genes, along with one control region (Table 2). The 37 encoded genes consist of 13 protein-coding genes (PCGs), 22 tRNA genes, and 2 rRNA genes. Most PCGs are located on the heavy strand (H-strand), with only the nad6 gene on the light strand (L-strand). Among the 22 tRNA genes, 14 are on the H-strand (*tRNA^Phe^*, *tRNA^Val^*, *tRNA^Leu(tta)^*, *tRNA^Ile^*, *tRNA^Met^*, *tRNA^Trp^*, *tRNA^Asp^*, *tRNA^Lys^*, *tRNA^Gly^*, *tRNA^Arg^*, *tRNA^His^*, *tRNA^Ser(gct)^*, *tRNA^Leu(tag)^*, *tRNA^Thr^*), and the remaining 8 are on the L-strand (*tRNA^Gln^*, *tRNA^Ala^*, *tRNA^Asn^*, *tRNA^Cys^*, *tRNA^Tyr^*, *tRNA^Ser(tga)^*, *tRNA^Glu^*, *tRNA^Pro^*) (Figure 2). These structural features are not only highly similar to those of other species in the genus *Liobagrus* but also consistent with the mitochondrial genome structures of *Amphilius* and even Cyprininae species, reflecting the stability of mitochondrial genomes in terms of structure and gene order [49,50].

### 3.2. Nucleotide Composition and Base Bias Analysis

The mitochondrial genome of *L. huaiheensis* exhibits an overall base composition characterized by A (30.87%) > T (24.87%) > C (28.85%) > G (15.41%). The A + T content accounts for 55.74%, significantly higher than the 44.26% G + C content. Additionally, the positive AT skew (0.10764) and negative GC skew (−0.30366) reflect a typical pattern of A-base preference and relative C-base enrichment (Table 3). Integrating comparative data of 13 *Liobagrus* species’ mitochondrial genomes (Table 4), the whole-genome A + T content (55.74%) of *L. huaiheensis* is moderately high within the genus (e.g., 56.19% in *L. stuarti*, 57.07% in *L. anguillicauda*). However, its GC skew (−0.3037) has a significantly higher absolute value than most species (e.g., −0.2797 in *L. marginatus*, −0.2796 in *L. mediadiposalis*), indicating stronger C-base enrichment. The PCGs’ A + T content (54.88%) aligns with the genus-wide average (53.44–56.73%), yet the more negative GC skew (−0.3264) suggests unique base composition in protein-coding regions. The rRNA genes’ A + T content (55.53%) shows minor differences from other genus species (54.08–56.74%), but the high AT skew (0.2401) reflects enhanced A-base preference in the rRNA region [51].

From the functional element perspective, most PCGs in *L. huaiheensis* show significant A-preference and C-enrichment. The *atp8* gene has a 37.58% A content and the highest genome-wide AT skew (0.26532), indicating strong A-preference. The light-strand *nad6* gene, due to strand localization, has a 40.74% T content (significantly higher than 14.04% A) and a negative AT skew (−0.48740), presenting a unique base distribution. At codon sites, the third site has the highest A (39.22%) and lowest G (7.41%) content, an extreme skew pattern related to mitochondrial energy metabolism functional requirements, potentially reflecting adaptive codon usage optimization for efficient transcription and translation [52,53,54]. tRNA genes have a 56.09% A + T content and a positive GC skew (0.03667, unique genome-wide), suggesting G-base preference, likely related to tRNA secondary structure stability [55]. The D-loop has the highest genome-wide A + T content (66.07%), with near-zero AT skew and negative GC skew, indicating high and balanced A/T content, associated with mitochondrial replication–transcription regulation [52]. The mitochondrial genome of *L. huaiheensis* presents core characteristics of high A + T content, A-base preference, and C-base enrichment. Compared to other *Liobagrus* species, it shows uniqueness in GC skew pattern and PCG base composition, reflecting functional adaptation in energy metabolism and genetic information transfer, and providing molecular evidence for resolving *Liobagrus* phylogenetic relationships.

### 3.3. Protein-Coding Genes and Codon Usage Characteristics

Among the 13 PCGs present in *L. huaiheensis*’ mitochondrial genome, ATG serves as the start codon for all, while *cox1* is an exception, initiating with GTG—a feature consistent with most fish species [56]. In contrast, stop codon usage is more diverse: *nad1*, *nad2*, *cox1*, and *nad6* employ the complete stop codon TAG; *atp8*, *atp6*, *nad4l*, and *nad5* use TAA as the complete stop codon; *cox3* has the incomplete stop codon TA-; and the remaining genes (*cox2*, *nad3*, *nad4*, and *cyt b*) terminate with the incomplete stop codon T- (Table 2). For genes with incomplete stop codons, mitochondria can dynamically form complete termination signals by adding adenines to the 3′ end of mRNA via polyadenylase, ensuring translational accuracy [57].

**Figure 3:**
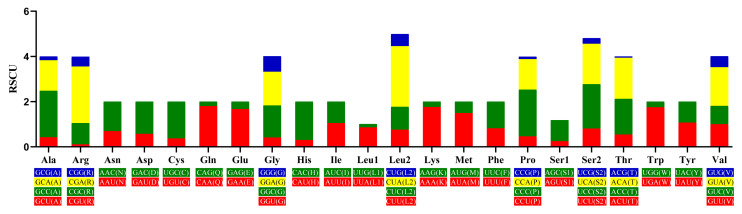
Relative synonymous codon usage (RSCU) of 13 PCGs in the mitogenome of *L. huaiheensis*.

**Figure 4:**
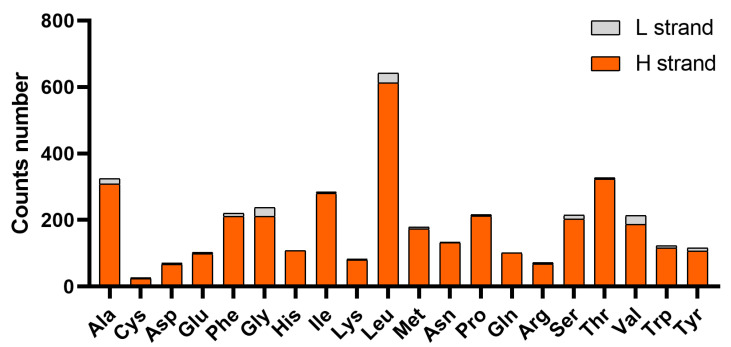
Mitochondrial genome codon usage of *L. huaiheensis*. Amino acids are displayed on the *X*-axis, and the *Y*-axis presents both the number of amino acids and gene direction. The H strand appears in orange, with the L strand in gray.

Statistics on codon counts and relative synonymous codon usage (RSCU) (Figure 3, Table 5, Figure 4) revealed that the preferred codons for most amino acids are NNA- or NNC-type, except for Tyr and Ile, which favor NNT-type codons—this differs significantly from the pattern observed in *Aloa lactinea* [58]. Additionally, the three codons with the highest RSCU values are CUA (RSCU = 2.68, encoding Leu), CGA (RSCU = 2.50, encoding Arg), and CCC (RSCU = 2.07, encoding Pro). In terms of amino acid usage frequency, Leu is the most abundant, followed by Ala and Thr; notably, the number of amino acids encoded by the heavy strand is significantly higher than that encoded by the light strand.

### 3.4. Characteristics of Ribosomal RNA and Transfer RNA Genes

**Figure 5:**
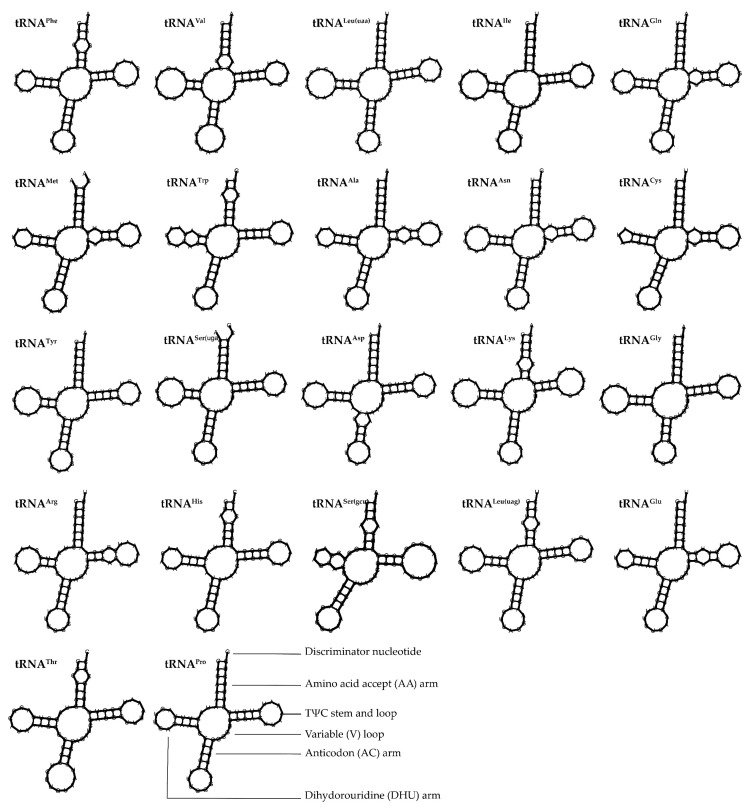
The secondary structure predictions of tRNA of *L. huaiheensis*.

In the mitochondrial genome of *L. huaiheensis*, the two ribosomal RNA (rRNA) genes located on the heavy strand, crucial for ribosome assembly and protein synthesis, are, respectively, transcribed into *12S rRNA*, with a length of 956 bp, and *16S rRNA*, which is 1666 bp in length. These rRNA genes exhibit a combined AT content of 55.54% and a pronounced A-base preference (AT skew = 0.24019). The encoded tRNA genes average approximately 71 bp in length, with *tRNA^Cys^* being the shortest (67 bp) and *tRNA^Leu(tta)^* the longest (75 bp) (Table 2 and Table 3). Collectively, the tRNA genes span 1560 bp, with an AT content of 56.09%—significantly lower than that reported for *Aloa lactinea* [58]. Notably, both the AT skew and GC skew of the tRNA genes in *L. huaiheensis* are positive, a characteristic distinct from the findings of Yang et al. in their study of *Arius maculatus* [59]. All tRNA genes can fold into the typical cloverleaf secondary structure (Figure 5). Specifically, there are eight pairs of unpaired bases in the amino acid acceptor arm, seven pairs of incorrectly paired bases in the TΨC stem and loop, and two pairs of unpaired bases in the DHU arm. The amino acid acceptor arm of all tRNAs is 6 bp in length, with the TΨC stem and loop ranging from 3 to 4 bp, the DHU arm from 2 to 3 bp, and the anticodon arm from 3 to 4 bp. Notably, the DHU arm of *tRNA^Cys^* exhibits an indistinct loop formation, and no obvious variable loop structure is observed in any of the tRNA secondary structures.

### 3.5. Selection Pressure Analysis

**Figure 6:**
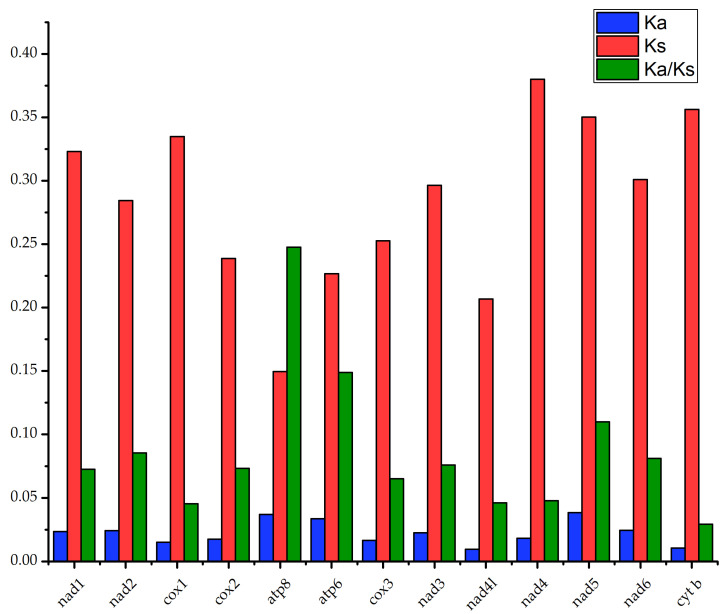
The Ka, Ks, and Ks/Ks values of 13 PCGs in *L. huaiheensis*.

To quantitatively assess the evolutionary significance of variable protein-coding sequences across divergent species, nonsynonymous (Ka) and synonymous (Ks) substitution rates are powerful metrics. The number of substitutions per nonsynonymous site is denoted by Ka, while that per synonymous site is denoted by Ks, and their ratio (Ka/Ks) classifies sequence evolution into three scenarios: negative (purifying) selection (Ka/Ks < 1), positive (adaptive) selection (Ka/Ks > 1), and neutral evolution (Ka/Ks = 1) [60,61,62]. The Ka, Ks, and Ka/Ks values of the 13 protein-coding genes in *L. huaiheensis* are presented in Figure 6. Genes with relatively high Ka values include *atp8* (0.0370), *atp6* (0.0337), and *nad5* (0.0384), whereas *nad4* exhibits the highest Ks value (0.3800). For Ka/Ks ratios, the *atp8* gene shows a significantly higher value (0.2476) compared to other genes, with the *cyt b* gene having the lowest (0.0293). Since all Ka/Ks ratios are less than 1, this indicates that all protein-coding genes in *L. huaiheensis* have undergone purifying selection, which helps maintain the stability of mitochondrial genes.

### 3.6. Results of Phylogenetic Analysis

**Figure 7:**
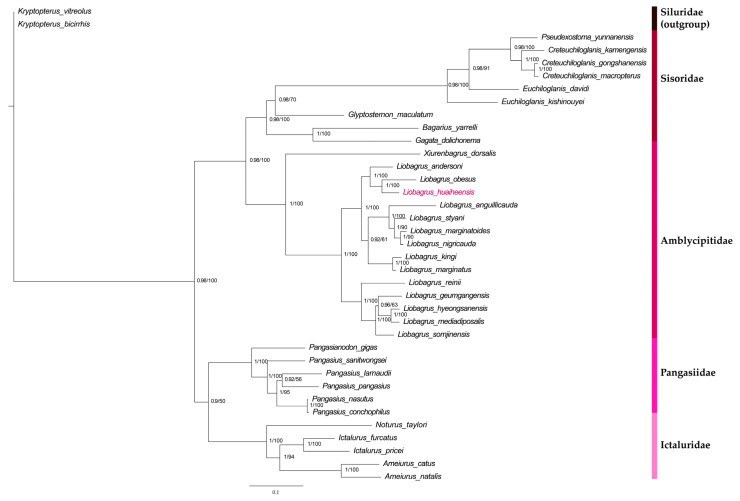
Phylogenetic analyses of *L. huaiheensis* based on 13 PCG nucleotide sequences from its mitogenome. Posterior probabilities from the BI and ML methods are shown on branch labels, and different families are distinguished by unique colors.

The optimal evolutionary models selected via PartitionFinder partitioning were as follows: the GTR + I + G model for *nad1*, *nad2*, *cox1*, *cox2*, *atp6*, *cox3*, *nad3*, *nad4*, *nad5*, and *cyt b*; and the GTR + G model for *atp8*, *nad4l*, and *nad6*. On the basis of these results, phylogenetic trees were constructed via BI and ML methods, using the nucleotide sequences of 13 PCGs derived from the mitochondrial genomes of 37 species (covering 14 genera and five families) (Figure 7). The trees showed that *L. huaiheensis* is most closely related to *L. obesus*, with a 100% posterior probability for this clade in both analyses. Based on the inferred tree topology, the currently recognized *Liobagrus* species can be clustered into three distinct clades, each with a posterior probability of 100%: (1) *L. huaiheensis*, *L. obesus*, and *L. andersoni*; (2) *L. anguillicauda*, *L. styani*, *L. marginatoides*, *L. nigricauda*, *L. kingi*, and *L. marginatus*; and (3) *L. reinii*, *L. geumgangensis*, *L. hyeongsanensis*, *L. mediadiposalis*, and *L. somjinensis*. The results of this study are consistent with those of Philjae Kim, who used unpartitioned mitochondrial genome data, and those of Chen Chongguang, who constructed trees based on partial *cyt b* sequences [24,63].

### 3.7. Population Genetic Diversity Analysis

Genetic diversity underpins a species’ survival, evolution, and adaptability to environmental alterations: typically, greater genetic diversity is associated with heightened adaptability and evolutionary potential. The *cox1* gene sequence, which exhibits high genetic polymorphism, has been widely utilized in studies on the genetic diversity of various fish species [64]. In our current study, we retrieved most of the *cox1* gene sequence from *L. huaiheensis*. After alignment and refinement, it reached a total length of 1150 bp, with only four distinct haplotypes identified. For the population of *L. huaiheensis* from the Huai River, the haplotype diversity (Hd) was 0.656 and the nucleotide diversity (π) was 0.00171. Both low values (Hd < 0.5 and π < 0.005, as shown in Table 6) suggest that this population may be at risk of genetic deterioration. Moreover, the results of Tajima’s D and Fu’s Fs tests indicated that this gene likely follows a neutral evolution model within the population, with no evident signals of strong impacts—such as natural selection or population expansion—on its genetic structure detected [65].

## 4. Conclusions

This study determined the complete mitochondrial genome of *L. huaiheensis*, a 16,512 bp circular molecule encoding 37 genes, with a conserved structure consistent with other *Liobagrus* species. Its mitogenome exhibits high A + T content (55.74%) with A-preference and C-enrichment, and unique base skew patterns in PCGs and tRNAs linked to functional adaptation. All 13 PCGs undergo purifying selection (Ka/Ks < 1), ensuring mitochondrial stability. Phylogenetic analyses (BI/ML) show *L. huaiheensis* is closest to *L. obesus* (100% support), with *Liobagrus* divided into three clades, consistent with prior studies. Population analysis of the *cox1* gene reveals low diversity (Hd = 0.656, π = 0.00171), indicating potential genetic deterioration, with neutral evolution supported by Tajima’s D and Fu’s Fs.

These results not only address existing data gaps regarding *L. huaiheensis* (a Huaihe endemic species), clarify its phylogenetic position within the genus, and provide foundational support for its conservation, but also lay a solid basis for subsequent research and applications. Specifically, they can be further leveraged to investigate the species’ geographical dispersal history, develop its DNA barcode for accurate species identification, explore the evolutionary mechanisms underlying its adaptation to specific environments, and screen potential genetic markers to facilitate marker-assisted breeding in aquaculture.

## Figures and Tables

**Table 1:** Detailed information of mitogenome sequences analyzed in this study, including NCBI accession numbers.

Family	Genus	Species	Genbank Accession No	Resource
Amblycipitidae	*Liobagrus*	*Liobagrus andersoni*	KX767082.1	[28]
		*Liobagrus anguillicauda*	JQ026256.1	Unpublished
		*Liobagrus geumgangensis*	NC_088753.1	[29]
		*Liobagrus hyeongsanensis*	MZ066608.1	[24]
		*Liobagrus kingi*	KC193779.1	[30]
		*Liobagrus marginatoides*	KC473938.1	[31]
		*Liobagrus marginatus*	KC757128.1	[32]
		*Liobagrus mediadiposalis*	KR075136.1	[33]
		*Liobagrus nigricauda*	KC316116.1	[34]
		*Liobagrus obesus*	JQ714035.1	[35]
		*Liobagrus somjinensis*	MN756661.1	[36]
		*Liobagrus styani*	KX096605.1	[37]
		*Liobagrus reinii*	AP012015.1	Unpublished
		*Liobagrus huaiheensis*	PV953861	This study
	*Xiurenbagrus*	*Xiurenbagrus dorsalis*	MN308285.1	Unpublished
Sisoridae	*Bagarius*	*Bagarius yarrelli*	JQ026260.1	[38]
	*Gagata*	*Gagata dolichonema*	JQ026250.1	Unpublished
	*Creteuchiloglanis*	*Creteuchiloglanis kamengensis*	MN396886.1	Unpublished
		*Creteuchiloglanis gongshanensis*	KP872697.1	Unpublished
		*Creteuchiloglanis macropterus*	KP872682.1	Unpublished
	*Euchiloglanis*	*Euchiloglanis davidi*	MK181572.1	[39]
		*Euchiloglanis kishinouyei*	JQ026252.1	Unpublished
	*Glyptosternon*	*Glyptosternon maculatum*	JQ026251.1	Unpublished
	*Pseudexostoma*	*Pseudexostoma yunnanensis*	JQ026258.1	Unpublished
Pangasiidae	*Pangasianodon*	*Pangasianodon gigas*	AY762971.1	[40]
	*Pangasius*	*Pangasius nasutus*	OQ078746.1	[41]
		*Pangasius sanitwongsei*	MN809630.1	[42]
		*Pangasius conchophilus*	OQ078745.1	[41]
		*Pangasius pangasius*	KC572135.1	Unpublished
		*Pangasius larnaudii*	AP012018.1	Unpublished
Siluridae	*Kryptopterus*	*Kryptopterus vitreolus*	KY710750.1	Unpublished
		*Kryptopterus bicirrhis*	KY569440.1	Unpublished
Ictaluridae	*Noturus*	*Noturus taylori*	KP013089.1	Unpublished
	*Ameiurus*	*Ameiurus catus*	MG570433.1	Unpublished
		*Ameiurus natalis*	MG570406.1	Unpublished
	*Ictalurus*	*Ictalurus furcatus*	KM576102.1	Unpublished
		*Ictalurus pricei*	KJ496298.1	[43]

**Table 2:** Annotation of the mitochondrial genome genes in *L. huaiheensis*.

Gene	Location		Strand	Gene Length (bp)	Intergenic Nucleotides	Overlapping Nucleotides	Codons	
	From	To					Start	Stop
*tRNA^Phe^*	1	69	H	69	-			
*12S rRNA*	70	1025	H	956				
*tRNA^Val^*	1026	1097	H	72				
*16S rRNA*	1098	2763	H	1666				
*tRNA^Leu(tta)^*	2764	2838	H	75				
*nad1*	2839	3810	H	972	5		ATG	TAG
*tRNA^Ile^*	3816	3887	H	72		1		
*tRNA^Gln^*	3887	3957	L	71		1		
*tRNA^Met^*	3957	4025	H	69				
*nad2*	4026	5072	H	1047		2	ATG	TAG
*tRNA^Trp^*	5071	5139	H	69	2			
*tRNA^Ala^*	5142	5210	L	69	1			
*tRNA^Asn^*	5212	5284	L	73	29			
*tRNA^Cys^*	5314	5380	L	67				
*tRNA^Tyr^*	5381	5451	L	71	1			
*cox1*	5453	7003	H	1551			GTG	TAG
*tRNA^Ser(tga)^*	7004	7074	L	71	4			
*tRNA^Asp^*	7079	7150	H	72	13			
*cox2*	7164	7854	H	691			ATG	T-
*tRNA^Lys^*	7855	7928	H	74	1			
*atp8*	7930	8097	H	168		10	ATG	TAA
*atp6*	8088	8771	H	684		1	ATG	TAA
*cox3*	8771	9555	H	785		1	ATG	TA-
*tRNA^Gly^*	9555	9627	H	73				
*nad3*	9628	9976	H	349			ATG	T-
*tRNA^Arg^*	9977	10,046	H	70				
*nad4l*	10,047	10,343	H	297		7	ATG	TAA
*nad4*	10,337	11,717	H	1381			ATG	T-
*tRNA^His^*	11,718	11,787	H	70				
*tRNA^Ser(gct)^*	11,788	11,856	H	69	2			
*tRNA^Leu(tag)^*	11,859	11,931	H	73				
*nad5*	11,932	13,755	H	1824		4	ATG	TAA
*nad6*	13,752	14,267	L	516			ATG	TAG
*tRNA^Glu^*	14,268	14,336	L	69	2			
*cyt b*	14,339	15,476	H	1138			ATG	T-
*tRNA^Thr^*	15,477	15,548	H	72		2		
*tRNA^Pro^*	15,547	15,616	L	70				
*CR*	15,617	16,512	H	896				

**Table 3:** Base composition characteristics of the mitogenome in *L. huaiheensis*.

Gene/Region	Size (bp)	Base Composition (%)						AT Skew	GC Skew
		A	T	C	G	A + T	G + C		
Genome	16,512	30.87	24.87	28.85	15.41	55.74	44.26	0.10764	−0.30366
PCGs	11,373	28.56	26.33	29.92	15.19	54.88	45.12	0.04069	−0.32645
*nad1*	969	27.97	25.39	32.92	13.73	53.36	46.65	0.04835	−0.41136
*nad2*	1044	32.95	22.51	32.28	12.26	55.46	44.54	0.18824	−0.44948
*cox1*	1548	26.16	28.10	28.23	17.51	54.26	45.74	−0.03575	−0.23437
*cox2*	690	30.87	26.09	27.97	15.07	56.96	43.04	0.08392	−0.29972
*atp8*	165	37.58	21.82	29.09	11.52	59.40	40.61	0.26532	−0.43265
*atp6*	681	32.01	24.96	29.66	13.36	56.97	43.02	0.12375	−0.37889
*cox3*	783	25.67	25.80	31.03	17.50	51.47	48.53	−0.00253	−0.27880
*nad3*	348	26.15	27.30	32.47	14.08	53.45	46.55	−0.02152	−0.39506
*nad4l*	294	23.47	24.49	35.03	17.01	47.96	52.04	−0.02127	−0.34627
*nad4*	1380	30.36	24.78	31.38	13.48	55.14	44.86	0.10120	−0.39902
*nad5*	1821	30.75	25.65	31.30	12.30	56.40	43.60	0.09043	−0.43578
*nad6*	513	14.04	40.74	9.94	35.28	54.78	45.22	−0.48740	0.56037
*cyt b*	1137	28.41	26.82	31.13	13.63	55.23	44.76	0.02879	−0.39097
First site	3791	27.64	20.18	27.20	24.98	47.82	52.18	0.15600	−0.04255
Secondary site	3791	18.81	40.60	27.41	13.19	59.41	40.60	−0.36677	−0.35025
Tertiary site	3791	39.22	18.20	35.16	7.41	57.42	42.57	0.36607	−0.65187
tRNA gene	1560	28.65	27.44	21.15	22.76	56.09	43.91	0.02157	0.03667
rRNA gene	2602	34.44	21.10	24.44	20.02	55.54	44.46	0.24019	−0.09942
D-loop zone	896	33.37	32.70	20.65	13.28	66.07	33.93	0.01014	−0.21721

**Table 4:** Comparative analysis of the complete mitochondrial genomes of 13 *Liobagrus* species.

Species	Whole Genome				PCGs				rRNA			
	Size	A + T	GC Skew	AT Skew	Size	A + T	GC Skew	AT Skew	Size	A + T	GC Skew	AT Skew
	(bp)	(%)			(bp)	(%)			(bp)	(%)		
*Liobagrus andersoni*	16,514	55.42	−0.2904	0.0962	11,373	54.63	−0.3085	0.0246	2620	55.11	−0.0986	0.2396
*Liobagrus anguillicauda*	16,536	57.07	−0.2813	0.0836	11,382	56.73	−0.3003	0.0150	2622	55.95	−0.0892	0.2243
*Liobagrus geumgangensis*	16,522	55.93	−0.2873	0.0894	11,377	55.10	−0.3019	0.0190	2624	55.34	−0.1058	0.2383
*Liobagrus hyeongsanensis*	16,529	55.41	−0.2806	0.0877	11,376	54.22	−0.2945	0.0156	2626	56.02	−0.1048	0.2373
*Liobagrus kingi*	16,483	54.10	−0.2822	0.0967	11,376	53.02	−0.2913	0.0211	2609	54.08	−0.1135	0.2417
*Liobagrus marginatoides*	16,498	55.67	−0.2870	0.0955	11,373	54.95	−0.3020	0.0186	2602	55.00	−0.0965	0.2383
*Liobagrus marginatus*	16,497	54.53	−0.2797	0.0944	11,376	53.47	−0.2942	0.0212	2570	54.09	−0.1000	0.2388
*Liobagrus mediadiposalis*	16,534	55.17	−0.2796	0.0907	11,376	53.89	−0.2901	0.0183	2626	55.98	−0.1038	0.2367
*Liobagrus nigricauda*	16,509	55.93	−0.2852	0.0929	11,371	55.29	−0.3025	0.0189	2618	55.42	−0.0968	0.2378
*Liobagrus obesus*	16,506	54.42	−0.2751	0.1069	11,376	53.46	−0.2920	0.0276	2619	55.25	−0.0973	0.2440
*Liobagrus somjinensis*	16,526	55.58	−0.2824	0.0883	11,376	54.34	−0.2980	0.0194	2624	56.10	−0.0972	0.2323
*Liobagrus styani*	16,515	56.19	−0.2886	0.0928	11,373	55.61	−0.3064	0.0212	2621	55.67	−0.0998	0.2378
*Liobagrus huaiheensis*	16,512	55.74	−0.3037	0.1076	11,373	54.88	−0.3264	0.0407	2602	55.53	−0.0994	0.2401

**Table 5:** Codon number and RSCU of 13 PCGs in the mitogenome of *L. huaiheensis*.

Amino Acid	Codon	Count	RSCU	Amino Acid	Codon	Count	RSCU
Phe	UUU	90	0.82	Tyr	UAU	62	1.07
Phe	UUC	130	1.18	Tyr	UAC	54	0.93
Leu	UUA	92	0.86	stop codon	UAA	4	1.00
Leu	UUG	16	0.15	stop codon	UAG	4	1.00
Leu	CUU	81	0.76	His	CAU	16	0.30
Leu	CUC	108	1.01	His	CAC	92	1.70
Leu	CUA	287	2.68	Gln	CAA	91	1.80
Leu	CUG	58	0.54	Gln	CAG	10	0.20
Ile	AUU	150	1.05	Asn	AAU	46	0.69
Ile	AUC	135	0.95	Asn	AAC	87	1.31
Met	AUA	133	1.49	Lys	AAA	72	1.76
Met	AUG	46	0.51	Lys	AAG	10	0.24
Val	GUU	53	1.00	Asp	GAU	20	0.57
Val	GUC	43	0.81	Asp	GAC	50	1.43
Val	GUA	91	1.71	Glu	GAA	86	1.67
Val	GUG	26	0.49	Glu	GAG	17	0.33
Ser	UCU	29	0.81	Cys	UGU	5	0.37
Ser	UCC	70	1.96	Cys	UGC	22	1.63
Ser	UCA	64	1.79	Trp	UGA	107	1.75
Ser	UCG	9	0.25	Trp	UGG	15	0.25
Pro	CCU	25	0.46	Arg	CGU	2	0.11
Pro	CCC	112	2.07	Arg	CGC	17	0.94
Pro	CCA	73	1.35	Arg	CGA	45	2.50
Pro	CCG	6	0.11	Arg	CGG	8	0.44
Thr	ACU	44	0.54	Ser	AGU	9	0.25
Thr	ACC	129	1.58	Ser	AGC	33	0.93
Thr	ACA	149	1.82	stop codon	AGA	0	0
Thr	ACG	5	0.06	stop codon	AGG	0	0
Ala	GCU	34	0.42	Gly	GGU	24	0.41
Ala	GCC	167	2.06	Gly	GGC	84	1.42
Ala	GCA	109	1.35	Gly	GGA	88	1.49
Ala	GCG	14	0.17	Gly	GGG	41	0.69

**Table 6:** *cox1* gene genetic diversity parameters in *L. huaiheensis* from the Huaihe River basin population.

Population	Gene	Number of Haplotypes	Haplotype (Gene) Diversity	Average Number of Nucleotide Difference	Nucleotide Diversity	Tajima’s D	Fu’s Fs
Huai River	*cox1*	4	0.656	1.888	0.00171	0.02195	1.899

## Data Availability

The data supporting the findings of this study are openly available in the National Center for Biotechnology Information (NCBI) database, accessible at https://www.ncbi.nlm.nih.gov (accessed on 22 July 2025) under the accession number PV953861.

## References

[1] Chen Z., Wu J., Wen A. (2021). *Liobagrus huaiheensis*, a New Species of Torrent Catfish (Teleostei: Siluriformes: Amblycipitidae) from the Huaihe River Basin in Central China. Zootaxa.

[2] Li Z., Huang R.J., Tian H.J., Wu K.J., Wang C.Z., Meng X.L., Zhou C.J., Gu Q.H., Nie G.X. (2015). Investigation of fishery resources in Xinyang Reaches of the Huai River. Henan Fish..

[3] Kim S.-H., Kim H.-S., Park J.-Y. (2015). A New Species of Torrent Catfish, *Liobagrus hyeongsanensis* (Teleostei: Siluriformes: Amblycipitidae), from Korea. Zootaxa.

[4] Sun Z.W., Ren S.J., Zhang E. (2013). *Liobagrus chenghaiensis*, a New Species of Catfish (Siluriformes: Amblycipitidae) from Yunnan, South China. Ichthyol. Exploit. Freshw..

[5] Xie R.-X., Zhang E. (2018). Re-Description of the Catfish Species *Liobagrus kingi* Tchang, 1935 (Pisces: Amblycipitidae) from the Upper Chang-Jiang Basin, China. Zootaxa.

[6] Roy A. (2014). Molecular Markers in Phylogenetic Studies—A Review. J. Phylogenetics Evol. Biol..

[7] Hurst G.D.D., Jiggins F.M. (2005). Problems with Mitochondrial DNA as a Marker in Population, Phylogeographic and Phylogenetic Studies: The Effects of Inherited Symbionts. Proc. R. Soc. B Biol. Sci..

[8] Duchêne S., Archer F.I., Vilstrup J., Caballero S., Morin P.A. (2011). Mitogenome Phylogenetics: The Impact of Using Single Regions and Partitioning Schemes on Topology, Substitution Rate and Divergence Time Estimation. PLoS ONE.

[9] Chen S., Zhou Y., Chen Y., Gu J. (2018). Fastp: An Ultra-Fast All-in-One FASTQ Preprocessor. Bioinformatics.

[10] Bankevich A., Nurk S., Antipov D., Gurevich A.A., Dvorkin M., Kulikov A.S., Lesin V.M., Nikolenko S.I., Pham S., Prjibelski A.D. (2012). SPAdes: A New Genome Assembly Algorithm and Its Applications to Single-Cell Sequencing. J. Comput. Biol..

[11] Nadalin F., Vezzi F., Policriti A. (2012). GapFiller: A de Novo Assembly Approach to Fill the Gap within Paired Reads. BMC Bioinform..

[12] Walker B.J., Abeel T., Shea T., Priest M., Abouelliel A., Sakthikumar S., Cuomo C.A., Zeng Q., Wortman J., Young S.K. (2014). Pilon: An Integrated Tool for Comprehensive Microbial Variant Detection and Genome Assembly Improvement. PLoS ONE.

[13] Birney E., Clamp M., Durbin R. (2004). GeneWise and Genomewise. Genome Res..

[14] Allio R., Schomaker-Bastos A., Romiguier J., Prosdocimi F., Nabholz B., Delsuc F. (2020). MitoFinder: Efficient Automated Large-Scale Extraction of Mitogenomic Data in Target Enrichment Phylogenomics. Mol. Ecol. Resour..

[15] Lowe T.M., Chan P.P. (2016). tRNAscan-SE On-Line: Integrating Search and Context for Analysis of Transfer RNA Genes. Nucleic Acids Res..

[16] Cui X., Lu Z., Wang S., Jing-Yan Wang J., Gao X. (2016). CMsearch: Simultaneous Exploration of Protein Sequence Space and Structure Space Improves Not Only Protein Homology Detection but Also Protein Structure Prediction. Bioinformatics.

[17] Griffiths-Jones S., Moxon S., Marshall M., Khanna A., Eddy S.R., Bateman A. (2005). Rfam: Annotating Non-Coding RNAs in Complete Genomes. Nucleic Acids Res..

[18] Donath A., Jühling F., Al-Arab M., Bernhart S.H., Reinhardt F., Stadler P.F., Middendorf M., Bernt M. (2019). Improved Annotation of Protein-Coding Genes Boundaries in Metazoan Mitochondrial Genomes. Nucleic Acids Res..

[19] Tamura K., Stecher G., Kumar S. (2021). MEGA11: Molecular Evolutionary Genetics Analysis Version 11. Mol. Biol. Evol..

[20] Choudhuri S., Sau K. (2024). CodonU: A Python Package for Codon Usage Analysis. IEEE/ACM Trans. Comput. Biol. Bioinform..

[21] Sahyoun A.H., Bernt M., Stadler P.F., Tout K. (2014). GC Skew and Mitochondrial Origins of Replication. Mitochondrion.

[22] Zhang Z. (2022). KaKs_Calculator 3.0: Calculating Selective Pressure on Coding and Non-Coding Sequences. Genom. Proteom. Bioinform..

[23] Larsson A. (2014). AliView: A Fast and Lightweight Alignment Viewer and Editor for Large Datasets. Bioinformatics.

[24] Kim P., Kim H., Kim S. (2021). Characterization of the Mitochondrial Complete Genome of Korean Indigenous Catfish, *Liobagrus hyeongsanensis* (Siluriformes: Amblycipitidae). Mitochondrial DNA Part B.

[25] Lanfear R., Frandsen P.B., Wright A.M., Senfeld T., Calcott B. (2017). PartitionFinder 2: New Methods for Selecting Partitioned Models of Evolution for Molecular and Morphological Phylogenetic Analyses. Mol. Biol. Evol..

[26] Minh B.Q., Schmidt H.A., Chernomor O., Schrempf D., Woodhams M.D., von Haeseler A., Lanfear R. (2020). IQ-TREE 2: New Models and Efficient Methods for Phylogenetic Inference in the Genomic Era. Mol. Biol. Evol..

[27] Ronquist F., Teslenko M., van der Mark P., Ayres D.L., Darling A., Höhna S., Larget B., Liu L., Suchard M.A., Huelsenbeck J.P. (2012). MrBayes 3.2: Efficient Bayesian Phylogenetic Inference and Model Choice Across a Large Model Space. Syst. Biol..

[28] Lee S., Kim J.H., Song H.Y. (2016). Complete Mitochondrial Genome of the Korean Torrent Catfish *Liobagrus andersoni* (Siluriformes, Amblycipitidae). Mitochondrial DNA Part B.

[29] Yun S., Park J. (2024). Characterization of the Complete Mitochondrial Genome of a Newly Discovered Torrent Catfish, *Liobagrus geumgangensis*, and Their Phylogenetic Relationships. Genes Genom..

[30] Jia X.-Y., Li Y.-W., Wang D.-Q., Tian H.-W., Xiong X., Li S.-H., Chen D.-Q. (2013). The Complete Mitochondrial Genome of *Liobagrus kingi* (Teleostei, Siluriformes: Amblycipitidae). Mitochondrial DNA.

[31] Jia X.-Y., Li Y.-W., Wang D.-Q., Tian H.-W., Tu B., Xiong X., Li S.-H., Chen D.-Q. (2013). The Mitogenome of *Liobagrus marginatoides* (Teleostei, Siluriformes:Amblycipitidae). Mitochondrial DNA.

[32] Li Q., Du J., Liu Y., Zhou J., Ke H., Liu C., Liu G. (2014). The Complete Mitochondrial Genome of *Liobagrus marginatus* (Teleostei, Siluriformes: Amblycipitidae). Mitochondrial DNA.

[33] Park C.E., Kim M.-C., Kim K.-H., Park H.C., Shin J.-H. (2017). The Complete Mitochondrial Genome Sequence of *Liobagrus mediadiposalis* (Teleostei, Siluriformes, Amblycipitidae). Mitochondrial DNA Part B.

[34] Jia X.-Y., Li Y.-W., Wang D.-Q., Li S.-H., Tian H.-W., Xiong X., Cheng X.-F., Chen D.-Q. (2013). The Mitogenome of *Liobagrus nigricauda* (Teleostei, Siluriformes: Amblycipitidae). Mitochondrial DNA.

[35] Kartavtsev Y.P., Jung S.-O., Lee Y.-M., Byeon H.-K., Lee J.-S. (2007). Complete Mitochondrial Genome of the Bullhead Torrent Catfish, *Liobagrus obesus* (Siluriformes, Amblycipididae): Genome Description and Phylogenetic Considerations Inferred from the Cyt *b* and 16S rRNA Genes. Gene.

[36] Kim P., Han J.-H., An S.L. (2020). Complete Mitochondrial Genome of Korean Catfish, *Liobagrus somjinensis* (Actinopterygii, Siluriformes, Amblycipitidae), from South Korea. Mitochondrial DNA Part B.

[37] Huang J.-Y., Hu S., Bai X., Zhang E. (2017). Complete Mitochondrial Genome of *Liobagrus styani* (Teleostei: Amblycipitidae). Mitochondrial DNA Part B.

[38] Du M., Zhou C.J., Niu B.Z., Liu Y.H., Li N., Ai J.L., Xu G.L. (2016). The Complete Mitochondrial Genome of the *Bagarius yarrelli* from Honghe River. IOP Conf. Ser. Earth Environ. Sci..

[39] Zou Y., Hu H., Zhang P., Wen Z., Wei Q. (2019). The Complete Mitochondrial Genome of Euchiloglanis Davidi and Its Phylogenetic Implications. Mitochondrial DNA Part B.

[40] Jondeung A., Sangthong P., Zardoya R. (2007). The Complete Mitochondrial DNA Sequence of the Mekong Giant Catfish (*Pangasianodon gigas*), and the Phylogenetic Relationships among Siluriformes. Gene.

[41] Halim S.A.A.A., Esa Y., Gan H.M., Zainudin A.A., Nor S.A.M. (2023). The Complete Mitochondrial Genomes of *Pangasius nasutus* and *P. conchophilus* (Siluriformes: Pangasiidae). Mitochondrial DNA Part B.

[42] Wei L., Ye X., Lv Y., Teng Z., Gan B., Zou H., Mo F., Zhang S. (2020). Complete Mitochondrial Genome and Phylogenetic Position of *Pangasius sanitwongsei* (Siluriformes: Pangasiidae). Mitochondrial DNA Part B.

[43] Ballesteros-Córdova C.A., Castañeda-Rivera M., Grijalva-Chon J.M., Castillo-Gámez R.A., Gutiérrez-Millán L.E., Camarena-Rosales F., Ruíz-Campos G., Varela-Romero A. (2016). Complete Mitochondrial Genome of *Ictalurus pricei* (Teleostei: Ictaluridae) and Evidence of a Cryptic Ictalurus Species in Northwest Mexico. Mitochondrial DNA Part A.

[44] Kuang T., Shuai F., Li X., Chen W., Lek S. (2021). Genetic Diversity and Population Structure of *Hemibagrus guttatus* (Bagridae, Siluriformes) in the Larger Subtropical Pearl River Based on COI and Cyt b Genes Analysis. Ann. Limnol. -Int. J. Lim..

[45] Chen Z.-G., Guo Y.-S., Dai Y.-T., Huang X.-C., Huang J.-H., Jiang J., Ouyang S., Wen A.-X., Wu X.-P. (2024). A New Species of *Liobagrus hilgendorf*, 1878 (Teleostei, Siluriformes, Amblycipitidae) from the Lower Changjiang River Basin in Southeast China. Zoosystematics Evol..

[46] Chen Z.-G., Guo Y.-S., Wu J.-Y., Wen A.-X. (2022). *Liobagrus chengduensis*, a New Species of Torrent Catfish (Teleostei: Siluriformes: Amblycipitidae) from the Upper Changjiang River Basin in Southwest China. Zool. Res..

[47] Clewley J.P. (1995). Macintosh Sequence Analysis Software. Mol. Biotechnol..

[48] Rozas J., Ferrer-Mata A., Sánchez-DelBarrio J.C., Guirao-Rico S., Librado P., Ramos-Onsins S.E., Sánchez-Gracia A. (2017). DnaSP 6: DNA Sequence Polymorphism Analysis of Large Data Sets. Mol. Biol. Evol..

[49] Zhang R., Zhu T., Li H., Deng L. (2023). The Mitochondrial Genome of *Linichthys laticeps* (Cypriniformes: Cyprinidae): Characterization and Phylogeny. Genes.

[50] Nakatani M., Miya M., Mabuchi K., Saitoh K., Nishida M. (2011). Evolutionary History of Otophysi (Teleostei), a Major Clade of the Modern Freshwater Fishes: Pangaean Origin and Mesozoic Radiation. BMC Evol. Biol..

[51] Grigoriev A. (1998). Analyzing Genomes with Cumulative Skew Diagrams. Nucleic Acids Res..

[52] Wolstenholme D.R., Wolstenholme D.R., Jeon K.W. (1992). Animal Mitochondrial DNA: Structure and Evolution. International Review of Cytology.

[53] Clayton D.A. (1982). Replication of Animal Mitochondrial DNA. Cell.

[54] Moritz C., Dowling T.E., Brown W.M. (1987). Evolution of Animal Mitochondrial DNA: Relevance for Population Biology and Systematics. Annu. Rev. Ecol. Syst..

[55] Burton Z.F. (2020). The 3-Minihelix tRNA Evolution Theorem. J. Mol. Evol..

[56] Yang T., Tan C., Zhao L., Hu Z., Su C., Li F., Ma Y., Zhang W., Hao X., Zou W. (2024). The Complete Mitochondrial Genome of the *Luciocyprinus langsoni* (Cypriniformes: Cyprinidae): Characterization, Phylogeny, and Genetic Diversity Analysis. Genes.

[57] Gagliardi D., Stepien P.P., Temperley R.J., Lightowlers R.N., Chrzanowska-Lightowlers Z.M.A. (2004). Messenger RNA Stability in Mitochondria: Different Means to an End. Trends Genet..

[58] Pan C., Xu S., Shu Y., Fang J. (2025). The Complete Mitochondrial Genome of Red Costate Tiger Moth (*Aloa lactinea* [Cramer, 1777]), and Phylogenetic Analyses of the Subfamily Arctiinae. Genes.

[59] Yang M., Yang Z., Liu C., Lee X., Zhu K. (2022). Characterization of the Complete Mitochondrial Genome of the Spotted Catfish *Arius maculatus* (Thunberg, 1792) and Its Phylogenetic Implications. Genes.

[60] Li J., Zhang Z., Vang S., Yu J., Wong G.K.-S., Wang J. (2009). Correlation Between Ka/Ks and Ks Is Related to Substitution Model and Evolutionary Lineage. J. Mol. Evol..

[61] Nekrutenko A., Makova K.D., Li W.-H. (2002). The K A/K S Ratio Test for Assessing the Protein-Coding Potential of Genomic Regions: An Empirical and Simulation Study. Genome Res..

[62] Zhang Z., Li J., Yu J. (2006). Computing Ka and Ks with a Consideration of Unequal Transitional Substitutions. BMC Evol. Biol..

[63] Chen Z. (2022). Taxonomic Study of *Liobagrus* (Siluriformes:Amblycipitidae) Destributed in Chinesemainland. Master’s Thesis.

[64] Gong J., Chen B., Li B., Zhou Z., Shi Y., Ke Q., Zhang D., Xu P. (2020). Genetic Analysis of Whole Mitochondrial Genome of *Lateolabrax maculatus* (Perciformes: Moronidae) Indicates the Presence of Two Populations along the Chinese Coast. Zoologia.

[65] Fu Y.-X. (1997). Statistical Tests of Neutrality of Mutations Against Population Growth, Hitchhiking and Background Selection. Genetics.

